# SNPchiMp: a database to disentangle the SNPchip jungle in bovine livestock

**DOI:** 10.1186/1471-2164-15-123

**Published:** 2014-02-11

**Authors:** Ezequiel Luis Nicolazzi, Matteo Picciolini, Francesco Strozzi, Robert David Schnabel, Cindy Lawley, Ali Pirani, Fiona Brew, Alessandra Stella

**Affiliations:** 1 Fondazione Parco Tecnologico Padano, Via Einstein, Loc. Cascina Codazza, Lodi 26900, Italy; 2 University of Missouri, Columbia, MO 65203, USA; 3 Illumina Inc, 5200 Illumina Way, San Diego, CA 92121, USA; 4 Affymetrix Inc, 3420 Central Expressway, Santa Clara, CA 95051, USA; 5 Affymetrix UK Ltd, Mercury Park, Wycombe Lane, High Wycombe HP10 0HH, UK; 6 Istituto di Biologia e Biotecnologia Agraria, Consiglio Nazionale delle Ricerche, Via Einstein, Cascina Codazza, Lodi 26900, Italy

**Keywords:** SNP chip, SNP data, Relational database, Assembly, Imputation, GWAS, Bovine livestock

## Abstract

**Background:**

Currently, six commercial whole-genome SNP chips are available for cattle genotyping, produced by two different genotyping platforms. Technical issues need to be addressed to combine data that originates from the different platforms, or different versions of the same array generated by the manufacturer. For example: i) genome coordinates for SNPs may refer to different genome assemblies; ii) reference genome sequences are updated over time changing the positions, or even removing sequences which contain SNPs; iii) not all commercial SNP ID’s are searchable within public databases; iv) SNPs can be coded using different formats and referencing different strands (e.g. A/B or A/C/T/G alleles, referencing forward/reverse, top/bottom or plus/minus strand); v) Due to new information being discovered, higher density chips do not necessarily include all the SNPs present in the lower density chips; and, vi) SNP IDs may not be consistent across chips and platforms. Most researchers and breed associations manage SNP data in real-time and thus require tools to standardise data in a user-friendly manner.

**Description:**

Here we present SNPchiMp, a MySQL database linked to an open access web-based interface. Features of this interface include, but are not limited to, the following functions: 1) referencing the SNP mapping information to the latest genome assembly, 2) extraction of information contained in dbSNP for SNPs present in all commercially available bovine chips, and 3) identification of SNPs in common between two or more bovine chips (e.g. for SNP imputation from lower to higher density). In addition, SNPchiMp can retrieve this information on subsets of SNPs, accessing such data either via physical position on a supported assembly, or by a list of SNP IDs, rs or ss identifiers.

**Conclusions:**

This tool combines many different sources of information, that otherwise are time consuming to obtain and difficult to integrate. The SNPchiMp not only provides the information in a user-friendly format, but also enables researchers to perform a large number of operations with a few clicks of the mouse. This significantly reduces the time needed to execute the large number of operations required to manage SNP data.

## Background

The public availability of many thousands of single nucleotide polymorphism (**SNP**) markers distributed across the genome has led to a new approach for genetic studies and applications [1]. SNPs are currently used for a wide range of analyses: in genomic association studies to dissect production and functional traits [2,3]; in prediction of (genomic) breeding values of livestock [4]; for estimation of genetic diversity and population genetic parameters [5] or to search for selection signatures in the genome [6]. Among livestock species, this technology has been applied most successfully in cattle, because factors such as evolutionary history, genetic structure, economics, etc. make cattle particularly suitable for the application of genome assisted selection.

Currently, six different SNP chips ranging from low to high density, on two different genotyping platforms, are available for cattle. Five SNP chips are available from Illumina (San Diego CA): Golden Gate Bovine3K BeadChip (Bovine3k), Infinium BovineLD BeadChip (BovineLD), Infinium BovineSNP50 v.1 BeadChip (BovineSNP50v.1), Infinium BovineSNP50 v.2 BeadChip (BovineSNP50v.2), Infinium BovineHD BeadChip (BovineHD), containing 2,900, 6,909, 54,001, 54,609 and 777,962 SNPs, respectively. Affymetrix has recently produced the Axiom Genome-Wide BOS 1 Bovine Array, containing 648,875 SNP probes (Axiom Bos 1). Considering the different chips and densities, and that data is routinely exchanged between projects and countries [4,7], there is the need for tools to effectively manage data and correlate information between SNP sets. Doing this necessitates maintaining updated information on the genome sequence (e.g. chromosome and position in base pairs) using the latest available assembly, and to accurately impute missing genotypes to obtain the highest value from the genotyping information [8,9].

Ambiguities in retrieving SNP information hinder the efficient use of data from different SNP panels in genomic applications. Researchers and breed associations routinely need to address several problems in order to manage data from different platforms, or even different versions of the same assay. For example, original reference genome sequences are updated over time, thus requiring updates to the genome coordinates for the SNPs on the chip(s); commercial SNP IDs might not be consistent within and across platforms; or SNP alleles might be coded using different formats and referencing strands.

Here we present SNPchiMp, a MySQL database linked to an open access web-based interface to manage bovine and eventually multi-species SNP chip data in a straightforward, flexible, consistent and user-friendly manner.

## Construction and content

### Data collection

SNP data were collected from three different sources. First, all bovine dbSNP database builds (http://www.ncbi.nlm.nih.gov/snp/) from June 2012 onward were downloaded to a local server (builds 136 and 137). The 29 bovine autosomal, sexual, mitochondrial, and unassigned chromosomes files were downloaded (~120 Gb). Second, Illumina SNP chip data were collected from manifest files of the Illumina GenomeStudio® software, and from data obtained directly from the manufacturer. The vast majority of this information is available directly from the Illumina website, “Products and Support files” section (http://support.illumina.com/array/downloads.ilmn). A total of 788,048 unique commercial SNP IDs (and linked information) were stored in the database, corresponding to all SNP IDs present in the five commercially available SNP chips plus an additional 4,275 cross-reference SNP IDs (e.g. same SNPs with different IDs). Associations between commercial SNP IDs and dbSNP rs/ss IDs for Illumina SNPs were first obtained by merging SNP IDs against the “Submitter ID” section in the previously downloaded dbSNP database. To confirm such associations, random sampled sequences were blasted against UMD3.1 assembly, and re-checked independently. The third source of information, the Affymetrix Axiom® Genome-Wide BOS 1 Array Plate (version 33), was obtained from the Affymetrix website support section (http://www.affymetrix.com/support/support_result.affx). This file contained 648,875 SNP probes, including 360,274 associations between SNP IDs and official rs IDs, thus no other integration of information was needed.

### Database architecture and web interface

**Figure 1:**
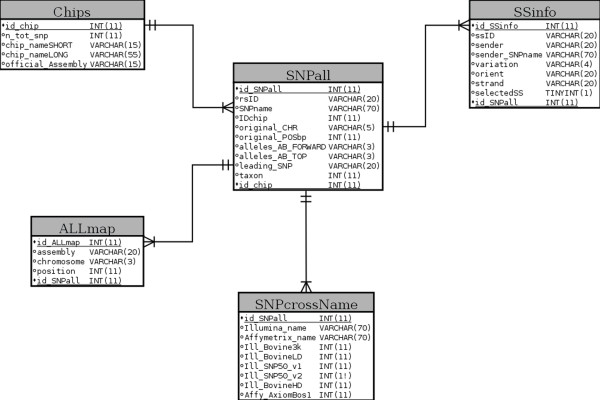
**E/R diagram of the SNPchiMp database.** Primary keys are evidenced with a black dot and underlined.

A MySQL relational database management system (RDBMS) was used to build and manage the underlying database in the SNPchiMp tool. The entity-relationship (E/R) diagram of the database is presented in Figure 1. A total of 12,171,260 records of SNP information, rs and ss IDs associations are currently available in the database. To manage the considerable volume of data stored into the database, ad-hoc optimisation strategies were developed. MySQL tables were indexed and logical partitions were created to decrease time and increase efficiency of the database queries. The Ruby programming language was used to code the web-based application. Given the proven stability and performance of the Java Virtual Machine (JVM), JRuby (http://www.jruby.org) was used as Ruby language interpreter and Ruby on Rails (RoR) version 3.2.11 (http://www.rubyonrails.org) was chosen as a web framework for a rapid development of the web interface and to handle the connection with the database. Finally, Torque Box (http://www.torquebox.org) was used as the application server to deploy the web service.

### Implementation of web-based tools

The base structure of the graphical interface was built using RoR base commands. A graphical template was designed using HTML and CSS languages, creating a common graphical layout for all the web pages in the graphical user interface (GUI). End users are able to interrogate the database in two dedicated sections: “Download” and “Browse”. Queries of the underlying database are facilitated through option selections using “check”, “select” and “input” boxes. Once the end user has selected the required options, the interrogation of the database is divided into two steps. First, a preview page displays the first 100 elements of the query. Only then, a second query allows the required data to be downloaded if the user selects the corresponding options. All files to be downloaded are automatically compressed, using a “tar” (i.e. tape archive) compression tool, in order to reduce download time.

## Utility

The SNPchiMp tool has six user menus: “Home”, “Manual”, “Download”, “Browse”, “About us” and “FAQs”. “Home” provides general information about the tool, “Manual”, contains a downloadable version of the manual, “About us” contains a short description of the people involved in the development of this tool, and “FAQs” are a list of frequently asked questions to solve specific issues not covered in the manual.

In the “Download” menu the user can download the information available in the database for one or more SNP chips. This menu is divided into two further sections: “Detailed SNP information” and “Across SNPchip table”. One of the main features of the “Detailed SNP information” menu allows conversion of mapping information (e.g. chromosome and position in base pairs) for one or more SNP chips to any of the available genome assemblies. Currently available assemblies are: native platform (e.g. original information in the genotyping platform producer files, independently from the assembly used for mapping SNPs); BTAU4.2 (map information extracted from dbSNP build 136); UMD3.1 and BTAU4.6 (map information extracted from dbSNP build 137). A “dummy” set of coordinates was assigned (e.g. chromosome 99 and position 0), for those loci where there was no commercial SNP ID - rs ID association (and for those SNPs whose rs ID were not mapped in the assembly considered; Table 1). In addition, users may download SNP ID, rs and ss ID associations (including linked information from dbSNP as sender names, strand, etc.), and allele coding to transform A/B allele coding into A/C/G/T forward or top format, and vice-versa. The “Across SNPchip table” section facilitates the download of a presence (coded as “1”)/absence (coded as “0”) table of SNPs across SNP chips. In addition, a list of common SNPs between SNP chips can be produced. The SNPs in common between all pairwise SNP chip combinations are shown in Table 2. To cover all possible combinations of data, associations between chips used the commercial SNP IDs for Illumina chips and rs IDs for associations between Illumina and Affymetrix SNP chips. All unique Illumina SNP IDs (even those with the same rs ID) are present. Cross-references for commercial SNP IDs were included for all chips by default; therefore, the number of rows in the files downloaded by the user may be larger than the number of SNPs in the referenced SNP chip (see values along the diagonal in Table 2). If required, Illumina SNP IDs may be associated with the corresponding Affymetrix SNP IDs using the rs ID to merge the records. In this case, two columns containing Illumina and Affymetrix SNP IDs are displayed (NULL data is shown for mismatching SNPs). For example, Illumina SNP IDs ‘ARS-USMARC-Parent-DQ647187-rs29010510’ and ‘BovineHD4000000047’ share the same rs ID ‘rs29010510’ with Affymetrix SNP ID ‘AX-24971295’. If both Illumina’s BovineHD and Affymetrix’s Axiom Bos 1 are chosen in this sub-section, when previewing the data, the user will obtain two rows with the Illumina SNP IDs in the column displaying Illumina SNP IDs and the (same) Affymetrix SNP ID in the column displaying Affymetrix SNP IDs.

**Table 1:** Consistency of information across SNP chip and assemblies

					**rs IDs without map information**^**e**^		
**SNPchip**	**Total**^**a**^	**no rs ID**^**b**^	**rsID ×2**^**c**^	**rsID ×3**^**d**^	**BTAU4.2**	**UMD3.1**	**BTAU4.6**
Bovine3k^f^	2,900	14	0	0	17	5	20
BovineLD^g^	6,909	3	0	1	94	14	111
BovineSNP50v.1^h^	54,001	0	29	0	1,970	204	1,852
BovineSNP50v.2^i^	54,609	18	29	0	2,154	340	2,031
BovineHD^j^	777,962	13	96	5	72,014	3,338	64,528
Axiom BOS 1^k^	648,875	288,601	1	0	130,618	0	16,207

^a^Total number of SNPs/SNP probes within chip not considering cross-references of SNP IDs.

^b^Total number of SNPs without SNP ID – rs ID association.

^c^Total number of SNPs with two different SNP IDs and same rs ID.

^d^Total number of SNPs with three different SNP IDs and same rs ID.

^e^Total number of SNPs whose rs ID is not mapped in the correspondent assembly (coded as chromosome 99 and position 0 in the SNPchiMp database).

^f^Illumina Golden Gate Bovine3K BeadChip.

^g^Illumina Infinium BovineLD BeadChip.

^h^Illumina Infinium BovineSNP50 v.1 BeadChip.

^i^Illumina Infinium BovineSNP50 v.2 BeadChip.

^j^Illumina Infinium BovineHD BeadChip.

^k^Affymetrix Axiom Genome-Wide BOS 1.

**Table 2:** Consensus information across SNP chips

	**Bov.3k**^**a**^	**Bov.LD**^**b**^	**Bov.SNP50v.1**^**c**^	**Bov.SNP50v.2**^**d**^	**Bov.HD**^**e**^	**Axiom BOS 1**^**f**^
**Bov.3k**	3,204 (304)					
**Bov.LD**	2,388 (229)	7,633 (724)				
**Bov.SNP50v.1**	3,190 (304)	7,589 (724)	58,276 (4,275)			
**Bov.SNP50v.2**	3,122 (298)	7,578 (724)	56,494 (4,154)	58,763 (4,154)		
**Bov.HD**	2,915 (275)	7,561 (717)	52,569 (3,835)	53,099 (3,754)	781,797 (3,835)	
**Axiom BOS 1**	1,861 (187)	4,510 (450)	38,527 (2,797)	38,376 (2,712)	87,398 (2,496)	648,875 (0)

^a^Illumina Golden Gate Bovine3K BeadChip.

^b^Illumina Infinium BovineLD BeadChip.

^c^Illumina Infinium BovineSNP50 v.1 BeadChip.

^d^Illumina Infinium BovineSNP50 v.2 BeadChip.

^e^Illumina Infinium BovineHD BeadChip.

^f^Affymetrix Axiom Genome-Wide BOS 1.

For across platform integration, rs IDs were used. Cross-referenced IDs are in parenthesis. Total number of SNP are displayed on the diagonal.

The “Browse” menu allows the user to query a custom list of SNPs by entering a list of commercial SNP IDs, rs IDs or ss IDs in the appropriate “input” boxes. It is also possible to browse SNPs by position in any of the available genome assemblies, using classical ENSEMBL chromosome:start:end syntax. Within this menu, users can preview the first 100 elements of the query. On selecting the desired SNP, the user may be re-directed to two external web pages (ENSEMBL and dbSNP), which contain specific SNP information. It should be noted that the information displayed in the external pages might be related to different genome assemblies than the version chosen by the user within the SNPchiMp tool. All the information from within SNPchiMP can be downloaded.

## Discussion

All the data used for building this tool are publicly available. Although this database includes information from commercial SNP chip platforms, these data can be freely obtained from the manufacturers publicly available web sites. The information in the database is not updated in real-time, but an in-house bioinformatics pipeline has been developed to ensure quick updates of the data when new releases of dbSNP and commercial chips become available.

The database was built using MySQL RDBMS as this is one of the most reliable open source database systems. Optimisation strategies and best practice translation rules were followed in order to enhance query performance of the web based GUI. The choice of JVM, the industry standard platform for web and applications development, combined with the flexibility of JRuby and Torque Box facilitated improvement in the performance of the SNPchiMp tool service (e.g. client–server request-answer time). RoR was chosen among other available options (e.g. PHP) as it allows fast development cycles and integration between Ruby code and html/javascript, increasing the flexibility of current and future integration of the database with other bioinformatic tools.

User interrogation of the database is carried out by selecting options from “check”, “select” or “input” boxes. This solution was chosen to enhance user-friendliness of the SNPchiMp tool. For this same reason, a three-step guided selection of options is displayed to the end user in the “Download” menu. This extra feature was added for clarity and to avoid input errors from the user. In order to avoid excessive downloads of large datasets, we employed a preview step of the first 100 elements of the query, only then allowing a second full query for download. This was an inexpensive solution as the wall-time required for previewing the data is negligible.

Ease of updating the map information for multiple SNP chips is one of the main features of this tool and allows users to readily update their chromosome and position information to any of the desired assemblies. Although this might seem trivial, it is in fact not a simple process for any of the non-model organisms and was one of the factors motivating the creation of this resource. In addition, this tool will be valuable to update SNP information as new genome assemblies are released. A centralized resource such as the SNPchiMp allows for the remapping, integration and updating of information to be carried out once and then easily accessed by the entire community. This resource will enable the community to use a standardized set of SNP information and minimize duplication of effort between labs. The other options, such as commercial SNP IDs and rs/ss IDs linked information can be used, for example, to merge genotypes of SNP chips of the same density but coded in different formats. This information is important for the integration (e.g. imputation) of high density SNP chip data to sequence data. The across SNP chip information was included to help users perform imputation of SNP data from lower to higher density panels and across platforms. In addition, combining this information with the most recent assembly (e.g. using the “Detailed SNP information” sub-section) can increase the accuracy of the imputation by reducing map errors.

The “Browse” menu allows researchers to browse genomic regions near specified SNPs, for example, significant SNPs from genome-wide association or selection sweep analyses. Although there is no direct link between this database and other publicly available databases, external links have been added in the preview menu to allow browsing for extra information using external resources.

## Conclusions

Dealing with whole genome SNP information currently requires that large amount of information are considered, coming from many different sources. The centralized database described herein gathers all this data and will hence reduce errors when integrating genomic information. The SNPChiMp database contains all commercial SNP panel information and rs ID (and ss ID) associations for all SNP chips available. In addition, the information contained in SNPchiMp can be integrated with other online databases, including those specific to livestock, such as SNAT (that includes only two SNP chips and is focused on functional SNPs) [10].

The current implementation of SNPchiMp was focused on integrating SNP information for cattle due to the number of assays currently available and multiple versions of genome assemblies. However, we recognized the utility of this tool across species and have built it in such a manner to allow inclusion of virtually any species with the same underlying data available. Both Illumina and Affymetrix SNP chip data are available for other species, including livestock, fish and companion animals; therefore, the database will be extended for these species in the future.

## Availability and requirements

SNPchiMp is freely available at http://bioinformatics.tecnoparco.org/SNPchimp. This tool is completely open source and multiplatform (accessible from any browser in any operating system). The information in this database is not updated in real-time. Considering that bovine SNP data is routinely updated, the Authors rely on feedback from the community to remain current.

## Abbreviations

SNP: Single nucleotide polymorphism; Bovine3k: Golden gate Bovine3K BeadChip; BovineLD: Illumina Infinium BovineLD BeadChip; BovineSNP50v.1: Illumina Infinium BovineSNP50 v.1 BeadChip; BovineSNP50v.2: Illumina infinium BovineSNP50 v.2 BeadChip; BovineHD: Illumina Infinium BovineHD BeadChip; Axiom Bos 1: Affymetrix Genome-Wide BOS 1 Bovine Array; RDBMS: Relational database management system; E/R: Entity-relationship; JVM: Java virtual machine; RoR: Ruby on Rails; GUI: Graphical user interface.

## Competing interests

Authors declare that they have no competing interests.

## Authors’ contributions

ELN, FS, AS conceived the study. ELN and MP built the database and the web-tool. RDS, CL, AP, FB provided the producer’s data, helped in the revision of the manuscript and helped in the improvement of functionality of the tool. RDS re-checked the database independently. ELN drafted the manuscript. All authors read and approved the final manuscript.
